# Convolution identities for divisor sums and modular forms

**DOI:** 10.1073/pnas.2322320121

**Published:** 2024-10-25

**Authors:** Ksenia Fedosova, Kim Klinger-Logan, Danylo Radchenko

**Affiliations:** ^a^Mathematical Institute, University of Münster, Münster 48149, Germany; ^b^Department of Mathematics, Kansas State University, Manhattan, KS 66506; ^c^Mathematical Department, University of Lille, Villeneuve d’Ascq Cedex 59655, France

**Keywords:** modular forms, convolution sums, *L*-values, graviton scattering

## Abstract

We consider certain convolution sums that are the subject of a conjecture by Chester, Green, Pufu, Wang, and Wen in string theory. We prove a generalized form of their conjecture, explicitly evaluating absolutely convergent sums

$$\sum_{n_{1}\in Z\setminus \left\{0,n\right\}}\varphi \left(n_{1},n-n_{1}\right)\sigma_{2m_{1}}\left(n_{1}\right)\sigma_{2m_{2}}\left(n-n_{1}\right),$$

where $\varphi (n_{1},n_{2})$ is a Laurent polynomial with logarithms. Contrary to original expectations, such convolution sums, suitably extended to $n_{1}\in \left\{0,n\right\}$, do not vanish, but instead, they carry number theoretic meaning in the form of Fourier coefficients of holomorphic cusp forms.

## Significance

Our research proves a conjecture from string theory asserting the vanishing of a specific convolution sum arising in the 4-graviton scattering amplitude in 10-dimensional type IIB string theory. The conjecture emerges from the Fourier expansion of solutions to a differential equation linked to the low-energy expansion of the scattering amplitude. Our work rigorously establishes this conjecture, showing the expected vanishing of the homogeneous solution. Moreover, our findings reveal a large family of convolution sums of a similar form that yield Fourier coefficients of modular forms.

In this paper we establish an identity giving a relationship between convolution sums of divisor functions $\sigma_{r}\left(n\right):=\sum_{d|n}d^{r}$ with $r\in 2Z_{\geq 0}$ and Fourier coefficients of Hecke eigenforms. For example, our main result implies that for *n* ≠ 0,

[1] $$\begin{array}{c} \sum_{\overset{n_{1},n_{2}\in Z\setminus \left\{0\right\}}{n_{1}+n_{2}=n}}\psi \left(n_{1},n_{2}\right)\sigma_{2}\left(n_{1}\right)\sigma_{2}\left(n_{2}\right)=-\left(\zeta \left(2\right)n^{2}+420\zeta^{\prime }\left(-2\right)\right)\sigma_{2}\left(n\right)-\frac{75L(\Delta ,6)\tau (|n|)}{{8L\left(\Delta ,5\right)|n|}^{3}}, \end{array}$$

where $\psi \left(n_{1},n_{2}\right)=\frac{1}{2}\left(\tilde{\psi }\left(n_{1},n_{2}\right)+\tilde{\psi }\left(n_{2},n_{1}\right)\right)$ for $\tilde{\psi }\left(n_{1},n_{2}\right)$ defined as

$$\begin{array}{c} \begin{array}{cc} \frac{1}{{\left(n_{1}+n_{2}\right)}^{3}} & \left(\frac{n_{1}^{5}}{n_{2}^{2}}+\frac{35n_{1}^{4}}{n_{2}}-1099n_{1}^{3}+1575n_{1}^{2}n_{2}+\left(420n_{1}^{3}-2100n_{1}^{2}n_{2}\right)\log\left|\frac{n_{1}}{n_{2}}\right|\right), \end{array} \end{array}$$

and L(Δ,s) is the *L*-function of the weight 12 cusp form $\Delta \left(z\right)=\sum_{n\geq 1}\tau \left(n\right)e^{2\pi \;inz}$. Such an identity is unexpected, and as far as the authors are aware, the only known relationship between divisor functions and the Ramanujan *τ* function involves finite sums of odd index divisor functions (1). Generally, shifted convolution sums are of number theoretic interest due to their connection to moments of and subconvexity bounds for *L*-functions (2–4). However, identities such as [**1**] would be difficult to discover outside of their natural context and, in this case, the investigation of the particular weighted sums is motivated by string theory.

Specifically, sums of the form Eq. **1** appear as part of the low energy expansion of the 4-graviton scattering amplitude as well as related calculations in the N=4 Super-Yang–Mills (SYM) gauge theory via the anti-de Sitter/conformal field theory correspondence (5–10). On the one hand, the appearance of holomorphic cusp forms in this context is unanticipated as they do not appear in corresponding localized computations (5). On the other hand, when computing the full integrated correlator, the exact identity established in *Theorem* 1 together with Manin’s Period Theorem allows one to see that these cusp forms exactly cancel. This cancellation suggests that the large-*N* expansion of certain integrals of the correlator of superconformal primary operators in the N=4 stress tensor multiplet can be written as lattice sums (5).

Our work was originally motivated by a conjecture from string theory of Chester, Green, Pufu, Wang, and Wen in (6, Section C.1(a)) that a particular shifted convolution sum vanishes, and *Theorem* 1 proves this conjecture. Explicitly, their conjecture can be written as

[2] $$\begin{array}{c} \begin{array}{c} \sum_{\overset{n_{1},n_{2}\in Z\setminus \left\{0\right\}}{n_{1}+n_{2}=n}}\varphi \left(n_{1},n_{2}\right)\sigma_{2}\left(n_{1}\right)\sigma_{2}\left(n_{2}\right)=\left(\frac{\zeta \left(2\right)n^{2}}{2}+30\zeta^{\prime }\left(-2\right)\right)\sigma_{2}\left(n\right), \end{array} \end{array}$$

where

[3] $$\begin{array}{cc} \varphi (n_{1},n_{2}) & =-\frac{n_{1}^{2}}{4n_{2}^{2}}-\frac{7n_{1}}{2n_{2}}-\frac{n_{2}^{2}}{4n_{1}^{2}}-\frac{7n_{2}}{2n_{1}}+\frac{47}{2}\\ & \;+\left(15-\frac{30n_{1}}{n_{1}+n_{2}}\right)\log\left|\frac{n_{1}}{n_{2}}\right|. \end{array}$$

Note that, unlike [**1**], there is no term involving Fourier coefficients of a cusp form in Eq. **2**. This conjecture arose from the fact that a constant multiple of the summation (plus the negation of the right side) in Eq. **2** appears in the homogeneous part of the Fourier expansion of a translation invariant solution to the equation

[4] $$\begin{array}{c} \left(\Delta -12\right)f\left(z\right)=-{\left(2\zeta \left(3\right)E_{3/2}\left(z\right)\right)}^{2}, \end{array}$$

where $\Delta =y^{2}\left(\partial_{x}^{2}+\partial_{y}^{2}\right)$ on ${\;\text{SL}}_{2}\left(Z\right)\backslash {\;\text{SL}}_{2}\left(R\right)/{\;\text{SO}}_{2}\left(R\right)=\Gamma \backslash H$ and $E_{s}\left(z\right)$ is the nonholomorphic Eisenstein series

$$\begin{array}{c} E_{s}\left(z\right)=\sum_{\gamma \in (B\cap \Gamma )\backslash \Gamma }\;\text{Im}{\left(\gamma z\right)}^{s},\;\;\text{for Re}\left(s\right)>1\; \end{array}$$

for *B* the Borel subgroup in ${\;\mathrm{SL}\;}_{2}\left(R\right)$ fixing $\infty \in \bar{H}$. Such solutions f(z) to Eq. **4** give the $D^{6}R^{4}$ coefficient of the low energy expansion of the 4-graviton scattering amplitude in 10-dimensional type IIB string theory (8–10). For physical reasons, it was expected that the homogenous solution should vanish.

In ref. 11, a formal argument was given for Eq. **2**; however, the argument relied on evaluating a double Dirichlet series outside its region of convergence, and the authors were unable to make this argument rigorous. Beyond looking for a rigorous proof of the conjecture, it is natural to ask whether other convolution sums similar to Eq. **2** might also hold. Explicitly, we can generate a family of convolution sums via computing homogeneous parts of the Fourier expansion of the solutions to differential equations of the form

[5] $$\begin{array}{c} \left(\Delta -s\left(s+1\right)\right)f\left(z\right)=E_{a}\left(z\right)E_{b}\left(z\right) \end{array}$$

for other values *a*, *b*, and *s* in R on Γ\H. Solutions $f(z)=E(s,a,b,z,\overset{¯}{z})$ to Eq. **5** are sometimes called generalized Eisenstein series and appear in calculations in N=4 SYM gauge theory (7) and physicists wondered whether the corresponding sums also vanish, giving identities similar to Eq. **2**. Numerical evidence told a surprisingly different story. In fact, instead of vanishing, these sums yield Fourier coefficients of modular forms as we will see in the statement of *Theorem* 1.

To state our main result precisely, recall the definition of Jacobi functions of the second kind [see (12, p. 172) for x∈C∖[−1,1], (13, section 4.61)]:

[6] $$\begin{array}{cc} Q_{d}^{\left(\alpha ,\beta \right)}\left(x\right) & =\frac{{\left(x-1\right)}^{-\alpha }{\left(x+1\right)}^{-\beta }}{2^{d+1}}\\ & \;\times \int_{-1}^{1}\frac{{\left(1-t\right)}^{d+\alpha }{\left(1+t\right)}^{d+\beta }dt}{{\left(x-t\right)}^{d+1}}, \end{array}$$

which we extend to x∈(−1,1) by setting $Q_{d}^{\left(\alpha ,\beta \right)}\left(x\right)=\frac{1}{2}\left(Q_{d}^{\left(\alpha ,\beta \right)}\left(x+i0\right)+Q_{d}^{\left(\alpha ,\beta \right)}\left(x-i0\right)\right)$. We will only discuss $Q_{d}^{\left(\alpha ,\beta \right)}$ for $\alpha ,\beta \in Z_{\geq 0}$ (more precisely, for $\alpha ,\beta \in 2Z_{\geq 0}$), in which case the expression on the right in Eq. **6** is single-valued in the cut plane C∖[−1,1] and defines an elementary function (see *Proposition* 1 below).

Theorem 1. *Let* $d\in Z_{>0}$ *and* $r_{1},r_{2}\in 2Z_{\geq 0}$. *Then, for any* $n\in Z_{>0}$,

[7] $$\begin{array}{cc} & \sum_{\begin{array}{c} n_{1},n_{2}\in Z\setminus \left\{0\right\}\\ n_{1}+n_{2}=n \end{array}}Q_{d}^{\left(r_{1},r_{2}\right)}(\frac{n_{2}-n_{1}}{n_{1}+n_{2}})\sigma_{r_{1}}\left(n_{1}\right)\sigma_{r_{2}}\left(n_{2}\right)\\ & \;={\left(-1\right)}^{d}Z_{d}^{\left(r_{1},r_{2}\right)}\left(n\right)\sigma_{r_{1}}\left(n\right)-Z_{d}^{\left(r_{2},r_{1}\right)}\left(n\right)\sigma_{r_{2}}\left(n\right)+\frac{a_{n}}{n^{d}}\;, \end{array}$$

*where*

$$\begin{array}{c} Z_{d}^{\left(\alpha ,\beta \right)}\left(n\right)=\left\{\begin{array}{cc} \frac{(\beta -1)!(\alpha +d)!}{2(\alpha +\beta +d)!}\zeta \left(\beta \right)n^{\beta }+\left(\begin{array}{c} d+\beta \\ d \end{array}\right)\frac{\zeta^{\prime }\left(-\beta \right)}{2}\;, & \beta \neq 0,\\ \frac{1}{4}\left(H_{d+\alpha }+H_{d}-\log\left|4\pi^{2}n\right|\right)\;, & \beta =0, \end{array}\right. \end{array}$$

*where *H*_*d*_ is the *d*-th harmonic number and* $h\left(\tau \right):=\sum_{m\geq 1}a_{m}q^{m}$ *is a cusp form of weight*

$$\begin{array}{c} k:=2d+r_{1}+r_{2}+2 \end{array}$$

*on* ${\mathrm{SL}}_{2}\left(Z\right)$, *given by* $h=\sum_{f}\lambda_{f}f$, where *f* runs over normalized Hecke eigenforms^*^ *of weight* *k* *and level* 1, *and*

$$\lambda_{f}=\frac{\pi {\left(-1\right)}^{d+r_{2}/2+1}}{2^{k}}\left(\begin{array}{c} k-2\\ d \end{array}\right)\frac{L^{⋆}\left(f,d+1\right)L^{⋆}\left(f,r_{1}+d+1\right)}{\left\langle f,f\right\rangle }\;.$$

*Here,* $\left\langle f,g\right\rangle :=\int_{\Gamma \backslash H}f\left(z\right)\bar{g(z)}y^{k-2}dxdy$ *denotes the Petersson inner product, and* $L^{⋆}\left(f,\cdot \right)$ *is the completed *L*-function of *f*:*

$$\begin{array}{c} L^{⋆}\left(f,s\right)={\left(2\pi \right)}^{-s}\Gamma \left(s\right)L\left(f,s\right). \end{array}$$

For *n* < 0, the identity [**7**] remains true if we write $\frac{a_{\left|n\right|}}{{\left|n\right|}^{d}}$ instead of $\frac{a_{n}}{n^{d}}$.

To see how this statement applies to solutions of differential equations of the form Eq. **5**, we note that for $a,b\in 1/2+Z_{>0}$ and *s* large enough, the homogeneous solution to Eq. **5** is given by

$$\begin{array}{c} \sum_{n\in Z}\alpha_{n}\sqrt{y}K_{s+1/2}\left(2\pi |n|y\right)e^{2\pi \;inx} \end{array}$$

for $K_{s}\left(z\right)$ the modified Bessel function of the second kind and *α*_*n*_ is a multiple of

$$\begin{array}{c} \begin{array}{cc} & \sum_{\begin{array}{c} n_{1},n_{2}\in Z\setminus \left\{0\right\}\\ n_{1}+n_{2}=n \end{array}}Q_{d}^{\left(r_{1},r_{2}\right)}(\frac{n_{2}-n_{1}}{n_{1}+n_{2}})\sigma_{r_{1}}\left(n_{1}\right)\sigma_{r_{2}}\left(n_{2}\right)\\ & \;\;+{\left(-1\right)}^{d+1}Z_{d}^{\left(r_{1},r_{2}\right)}\left(n\right)\sigma_{r_{1}}\left(n\right)+Z_{d}^{\left(r_{2},r_{1}\right)}\left(n\right)\sigma_{r_{2}}\left(n\right), \end{array} \end{array}$$

where $r_{1}=2a-1$, $r_{2}=2b-1$, and d=s+1−a−b (see ref. 14).

In what remains of the introduction, we will discuss other work related to convolution sums of divisor functions. In Section 1.2, we give a corollary of *Theorem* 1 which expresses [**7**] in terms of polynomials and logarithms as opposed to Jacobi functions of the second kind. We will also provide some examples of identities of the form in Eq. **7** and discuss the ramifications of *Theorem* 1 in physics. In Section 2, we will establish properties of the Jacobi function with integer parameters which we later use in the proof of our main result. In Section 3, we will first prove some integral identities involving Whittaker functions. We then provide a precise statement of the Holomorphic Projection Lemma which we will then use to prove *Theorem* 1.

### 1.1. Related Results.

Before the full theory of modular forms had been developed, Jacobi, Glaisher (15), and Ramanujan (1) examined sums involving divisor functions. The formula

[8] $$\begin{array}{c} \sum_{n=1}^{N-1}\sigma_{3}\left(n\right)\sigma_{3}\left(N-n\right)=\frac{1}{120}\left(\sigma_{7}\left(N\right)-\sigma_{3}\left(N\right)\right) \end{array}$$

was attributed to Jacobi^†^ (16). In 1885, Glaisher gave expressions for the first powers of series where the coefficients are the sums of divisor function (15). Motivated by generalizing [**8**], Ramanujan manipulated what would later be known as the Eisenstein series $E_{2},E_{4},$ and *E*_6_ to study finite convolution sums of odd divisor functions (1, pp. 136–162). Later in 1969, Lahiri found identities involving sums of *σ*_1_ shifted by pentagonal numbers (17).

Generally, identities similar to Eq. **8** involving finite convolution sums of odd divisor functions can be found using holomorphic Eisenstein series by computing the Fourier coefficients of their products or Rankin-Cohen brackets (18, pp. 18 and 56). Many other examples of such formulas were derived in a recent work of O’Sullivan (19) using holomorphic projection. However, none of these identities treat even index divisor functions nor infinite sums of them as in Eq. **7**.

Perhaps more closely related to this work, Diamantis proved that one can express quotients of values of *L*-functions associated to a normalized cusp form in terms of certain shifted convolution sums (20, *Theorem* 1.1). The formula in Eq. **7** gives an explicit form of such an expression. Motohashi gives a representation in terms of spectral data of a weighted sum of divisor functions $\sum_{n=1}^{\infty }\sigma_{0}\left(n\right)\sigma_{0}\left(n+f\right)W\left(n/f\right)$, where *f* ≥ 1 and $W\;\in \;C_{0}^{\infty }\left(R_{>0}\right)$ (16, *Theorem* 3).

### 1.2. Corollaries and Applications.

As they appear in string theory, it is not obvious that these divisor sums can be expressed in terms of Jacobi functions. It may be useful to instead think of these weightings as combinations of polynomials in *n*_*j*_, $1/n_{j},$ and $\log|n_{j}|$ for j=1,2.

Corollary 1. *For* $r_{1},r_{2}\in 2Z_{\geq 0}$, $d\in Z_{>0}$ *and* *n* *> 0, let*

[9] $$\begin{array}{c} \begin{array}{cc} \varphi (n_{1},n_{2}) & :=\sum_{j=-r_{1}}^{d-1}A_{j}n_{1}^{j}+\sum_{j=-r_{2}}^{d-1}B_{j}n_{2}^{j}\\ & \;\;+\sum_{j=0}^{d}\left(C_{j}n_{1}^{j}\log|n_{1}|+D_{j}n_{2}^{j}\log\left|n_{2}\right|\right) \end{array} \end{array}$$

*be such that* $\varphi \left(n_{1},n-n_{1}\right)=O\left(n_{1}^{-d-r_{1}-r_{2}-1}\right)$ *for* $n_{1}\to \pm \infty$. *Then, for* $n_{1}+n_{2}=n$,

[10] $$\begin{array}{c} \varphi \left(n_{1},n_{2}\right)=\Gamma_{d}^{\left(r_{1},r_{2}\right)}Q_{d}^{\left(r_{1},r_{2}\right)}\left(\frac{n_{2}-n_{1}}{n_{2}+n_{1}}\right) \end{array}$$

*and thus*

$$\begin{array}{c} \begin{array}{cc} & \sum_{\begin{array}{c} n_{1}+n_{2}=n\\ n_{1}n_{2}\neq 0 \end{array}}\varphi \left(n_{1},n_{2}\right)\sigma_{r_{1}}\left(n_{1}\right)\sigma_{r_{2}}\left(n_{2}\right)=\Gamma_{d}^{\left(r_{1},r_{2}\right)}\\ & \;\;\;\;\;\times \left[{\left(-1\right)}^{d}Z_{d}^{\left(r_{1},r_{2}\right)}\left(n\right)\sigma_{r_{1}}\left(n\right)-Z_{d}^{\left(r_{2},r_{1}\right)}\left(n\right)\sigma_{r_{2}}\left(n\right)+\frac{a_{n}}{n^{d}}\right], \end{array} \end{array}$$

*where* $Z_{d}^{\left(\alpha ,\beta \right)}$ *and *a*_*n*_ are as defined in *Theorem* 1, and*

[11] $$\begin{array}{c} \Gamma_{d}^{\left(r_{1},r_{2}\right)}:={\left(-1\right)}^{d+1}C_{d}n^{d}\frac{2d!(r_{1}+r_{2}+d)!}{(r_{1}+r_{2}+2d)!}, \end{array}$$

*where* *C*_*d*_ *is as in* Eq. **9**.

Note that the values of the constants *A*_*j*_, *B*_*j*_, *C*_*j*_, and *D*_*j*_ are fixed by Eqs. **10** and **11**.

For $r_{1}=r_{2}=0$ or $r_{1}r_{2}\neq 0$, the first two terms on the right-hand side of Eq. **7** coincide with the ones predicted formally in ref. 21. In the case when there are no cusp forms of weight $k:=2d+r_{1}+r_{2}+2$, the predictions given by the formal arguments in refs. 11 and 21 are proven by *Theorem* 1. Explicitly, when specifying $r_{1}=r_{2}=2$ and *d* = 1, since there are no cusp forms of weight 8, *Corollary* 1 proves the identity [**2**] as originally conjectured in ref. 6. Moreover, *Corollary* 1 proves the conjectures in ref. 21 by showing that when $r_{1}=r_{2}=0$ and *d* = 1, we have

[12] $$\begin{array}{c} \begin{array}{cc} & \sum_{\overset{n_{1},n_{2}\in Z\setminus \left\{0\right\}}{n_{1}+n_{2}=n}}\sigma_{0}\left(n_{1}\right)\sigma_{0}\left(n_{2}\right)[\frac{n_{2}-n_{1}}{n}\log\left|\frac{n_{1}}{n_{2}}\right|+2]\\ & \;\;=\left(2-\log\left(4\pi^{2}\left|n\right|\right)\right)\sigma_{0}\left(n\right), \end{array} \end{array}$$

and, when $r_{1}=r_{2}=0$ and *d* = 3, we get that

$$\begin{array}{c} \begin{array}{cc} & \sum_{\overset{n_{1},n_{2}\in Z\setminus \left\{0\right\}}{n_{1}+n_{2}=n}}\sigma_{0}\left(n_{1}\right)\sigma_{0}\left(n_{2}\right)\psi_{1}\left(n_{1},n_{2}\right)\\ & \;\;=\left(11-3\log(4\pi^{2}|n|)\right)\sigma_{0}\left(n\right), \end{array} \end{array}$$

where $\psi_{1}\left(n_{1},n_{2}\right)$ equals

$$\begin{array}{c} \begin{array}{c} 11-\frac{60n_{1}n_{2}}{n^{2}}-\frac{3n_{1}^{3}-27n_{1}^{2}n_{2}+27n_{1}n_{2}^{2}-3n_{2}^{3}}{n^{3}}\log\left|\frac{n_{1}}{n_{2}}\right| \end{array} \end{array}$$

for $n=n_{1}+n_{2}$.

Furthermore, when analyzing the homogeneous solution to Eq. **5** for a=b=3/2 and *s* = 5, a constant multiple of

$$\begin{array}{c} \sum_{\overset{n_{1},n_{2}\in Z\setminus \left\{0\right\}}{n_{1}+n_{2}=n}}\psi \left(n_{1},n_{2}\right)\sigma_{2}\left(n_{1}\right)\sigma_{2}\left(n_{2}\right) \end{array}$$

for *ψ* as in Eq. **2** appears. Using *Corollary* 1 when $r_{1}=r_{2}=2$ and *d* = 3, one sees that [**1**] holds. As a final example, in the homogeneous solution to Eq. **5** for a=3/2, b=5/2, and *s* = 11 involves a constant multiple of the sum

[13] $$\begin{array}{c} \sum_{\overset{n_{1},n_{2}\in Z\setminus \left\{0\right\}}{n_{1}+n_{2}=n}}\sigma_{2}\left(n_{1}\right)\sigma_{4}\left(n_{2}\right)\psi_{2}\left(n_{1},n_{2}\right), \end{array}$$

where

$$\begin{array}{cc} \psi_{2}\left(n_{1},n_{2}\right) & =\frac{7106n_{1}^{7}}{n^{7}}-\frac{22287n_{1}^{6}}{n^{6}}+\frac{84626n_{1}^{5}}{3n^{5}}-\frac{110789n_{1}^{4}}{6n^{4}}\\ & \;+\frac{33286n_{1}^{3}}{5n^{3}}-\frac{3893n_{1}^{2}}{3n^{2}}+\frac{2614n_{1}}{21n}-\frac{1727}{420}+\frac{n}{63n_{1}}\\ & \;+\frac{n^{2}}{8190n_{1}^{2}}-\frac{22n}{63n_{2}}-\frac{11n^{2}}{1365n_{2}^{2}}-\frac{n^{3}}{4095n_{2}^{3}}-\frac{n^{4}}{180180n_{2}^{4}}\\ & \;-(\frac{11n_{1}^{8}}{n^{8}}-\frac{176n_{1}^{7}n_{2}}{n^{8}}+\frac{924n_{1}^{6}n_{2}^{2}}{n^{8}}-\frac{2112n_{1}^{5}n_{2}^{3}}{n^{8}}+\frac{2310n_{1}^{4}n_{2}^{4}}{n^{8}}\\ & \;-\frac{1232n_{1}^{3}n_{2}^{5}}{n^{8}}+\frac{308n_{1}^{2}n_{2}^{6}}{n^{8}}-\frac{32n_{1}n_{2}^{7}}{n^{8}}+\frac{n_{2}^{8}}{n^{8}})\log|\frac{n_{1}}{n_{2}}|. \end{array}$$

Using $r_{1}=2$, $r_{2}=4$, and *d* = 8 in *Corollary* 1, we get that [**13**] is equal to

$$\begin{array}{c} \left(\frac{\zeta (4)}{180180}n^{4}+\frac{33\zeta (5)}{4\pi^{4}}\right)\sigma_{2}\left(n\right)+\left(-\frac{\zeta (2)}{8190}n^{2}+\frac{\zeta (3)}{4\pi^{2}}\right)\sigma_{4}\left(n\right)+\frac{a_{n}}{n^{8}}, \end{array}$$

where

$$\begin{array}{c} \begin{array}{cc} \sum_{n\geq 1}a_{n}e^{2\pi \;inz} & =\frac{L(9,f_{1})}{168L(8,f_{1})}\left(-29+\frac{3551}{\sqrt{144169}}\right)f_{1}\left(z\right)\\ & \;+\frac{L(9,f_{2})}{168L(8,f_{2})}\left(-29-\frac{3551}{\sqrt{144169}}\right)f_{2}\left(z\right) \end{array} \end{array}$$

and

$$\begin{array}{cc} f_{1}\left(z\right) & =e^{2\pi \;iz}+\left(540-12\sqrt{144169}\right)e^{4\pi \;iz}+\cdots \\ f_{2}\left(z\right) & =e^{2\pi \;iz}+\left(540+12\sqrt{144169}\right)e^{4\pi \;iz}+\cdots \end{array}$$

are normalized Hecke eigenforms of weight 24.

One implication of *Theorem* 1 is that certain linear combinations of generalized Eisenstein series $E(s,a,b,z,\overset{¯}{z})$ arising in physics have no cuspidal components. Such linear combinations of generalized Eisenstein series occur when examining the regularized large *N* expansion of certain integrated correlators in SU(N)N=4 SYM theory. Specifically, it was recently understood that the four superconformal primary operators in the N=4 stress tensor multiplet are obtained from derivatives of the partition *Z* of the mass-deformed SU(N)N=4 SYM theory placed on a squashed four-sphere (22, 23). For the partition function *Z*, the $N^{-3}$-term of $\partial_{m}^{4}{\log Z|}_{m=0,b=1}$ (6, section 2.13) is given by

[14] $$\begin{array}{c} \begin{array}{cc} \alpha_{3}\; & E\left(3,\frac{3}{2},\frac{3}{2},z,\overset{¯}{z}\right)+\sum_{r=5,7,9}\left[\alpha_{r}\;E\left(r,\frac{3}{2},\frac{3}{2},z,\overset{¯}{z}\right)\right.\\ & \left.+\beta_{r}\;E\left(r,\frac{5}{2},\frac{5}{2},z,\overset{¯}{z}\right)+\gamma_{r}\;E\left(r,\frac{7}{2},\frac{3}{2},z,\overset{¯}{z}\right)\right], \end{array} \end{array}$$

where $\alpha_{i},\beta_{i}$ and *γ*_*i*_ are all constants defined in ref. 6, section 2.14.

When *r* = 5, we expect the terms in the Fourier expansions of $E\left(r,\frac{3}{2},\frac{3}{2},z,\overset{¯}{z}\right)$, $E\left(r,\frac{5}{2},\frac{5}{2},z,\overset{¯}{z}\right),$ and $E\left(r,\frac{7}{2},\frac{3}{2},z,\overset{¯}{z}\right)$ which correspond to the homogeneous solution to Eq. **5** will contain *L*-values and Fourier coefficients of the weight 12 cusp form^‡^ Δ. However, the linear combination of these terms appearing in Eq. **14** vanishes. To see this, let $L^{⋆}\left(s\right):=L^{⋆}\left(\Delta ,s\right)$ and L(s):=L(Δ,s) and note that ⟨Δ,Δ⟩ is a common denominator in all terms, and thus, we can omit it from the consideration. Moreover, the $Z_{d}^{\left(r_{1},r_{2}\right)}$ terms will vanish as they simply contribute to the cases when $n_{1}n_{2}=0$. Thus, it suffices to consider the sum

$$\begin{array}{c} \frac{4032}{5\pi^{4}}\alpha_{5}D\left(2,2,3\right)+\frac{7168}{5\pi^{2}}\beta_{5}D\left(4,4,1\right)+\frac{3072}{5\pi^{2}}\gamma_{5}D\left(2,6,1\right), \end{array}$$

for

$$\begin{array}{c} \begin{array}{cc} D(r_{1},r_{2},d) & :={\left(-1\right)}^{d+\frac{r_{2}}{2}+1}2^{-(2d+r_{1}+r_{2}+2)}\\ & \;\;\times L^{⋆}\left(d+1\right)L^{⋆}\left(d+r_{1}+1\right)\left(\begin{array}{c} 2d+r_{1}+r_{2}\\ d \end{array}\right) \end{array} \end{array}$$

and $\alpha_{5}=-\frac{135}{52\pi^{3}}$, $\beta_{5}=-\frac{30375}{832\pi^{5}}$, and $\gamma_{5}=-\frac{42525}{832\pi^{5}}$ (6, section 2.14). After a substitution, it suffices to check that

$$\begin{array}{c} \begin{array}{c} -\frac{382725L(2)L(4)}{53248\pi^{13}}-\frac{1148175L(4)L(6)}{26624\pi^{17}}+\frac{3189375L(2)L(6)}{53248\pi^{15}} \end{array} \end{array}$$

vanishes, which can be done with the help of ref. 24.

We note that the cuspidal contribution to Eq. **14** also vanishes when *r* = 7 and *r* = 9 confirming physical heuristics. In fact, in ref. 5, the authors found that for higher-order terms in the 1/N expansion for the integrated correlator, the cuspidal terms cancel as in ref. **14**, implying that these terms can be represented as lattice sums. This result further suggests that there should be a more optimal choice than generalized Eisenstein series as a basis for such computations.

## 2. Jacobi Functions with Integer Parameters

We note that in the case $\alpha ,\beta ,d\in Z_{\geq 0}$, the Jacobi functions $Q_{d}^{\left(\alpha ,\beta \right)}\left(x\right)$ can be expressed in elementary terms and have the following characterization (compare with (25, equation 5.7)).

Proposition 1. *Let* $\alpha ,\beta ,d\in Z_{\geq 0}$ *and let* $P_{d}^{\left(\alpha ,\beta \right)}$ *denote the Jacobi polynomial defined in ref.* *13**, section 4.1. For* x∈R∖{−1,1}, *we have*

[15] $$\begin{array}{c} Q_{d}^{\left(\alpha ,\beta \right)}\left(x\right)=\frac{{\left(-1\right)}^{\alpha }}{2}P_{d}^{\left(\alpha ,\beta \right)}\left(x\right)\log|\frac{x+1}{x-1}|+\frac{R(x)}{{\left(x-1\right)}^{\alpha }{\left(x+1\right)}^{\beta }}, \end{array}$$

*where* R∈Q[x] *is a polynomial of degree* d+α+β−1. *Moreover, let *F* be any function of the form*

[16] $$\begin{array}{c} F\left(x\right)=P\left(x\right)\log|\frac{x+1}{x-1}|+\frac{R(x)}{{\left(x-1\right)}^{\alpha }{\left(x+1\right)}^{\beta }} \end{array}$$

*with* P,R∈R[x], *and* *P* *of degree* ≤d *such that* $F\left(x\right)=O\left(x^{-d-\alpha -\beta -1}\right)$, x→∞*; then, *F* must be a multiple of* $Q_{d}^{\left(\alpha ,\beta \right)}\left(x\right)$.

**Proof**: The first claim follows from the integral representation (13, equation 4.61.4)

$$\begin{array}{c} \begin{array}{cc} Q_{d}^{\left(\alpha ,\beta \right)}\left(x\right) & =\frac{{\left(x-1\right)}^{-\alpha }{\left(x+1\right)}^{-\beta }}{2}\\ & \;\times \int_{-1}^{1}\frac{{\left(1-t\right)}^{\alpha }{\left(1+t\right)}^{\beta }P_{d}^{\left(\alpha ,\beta \right)}\left(t\right)dt}{\left(x-t\right)}\;, \end{array} \end{array}$$

and writing $\int_{-1}^{1}\frac{p(t)dt}{x-t}=p\left(x\right)\int_{-1}^{1}\frac{\mathrm{dt}}{x-t}-\int_{-1}^{1}\frac{p(x)-p(t)}{x-t}dt$. From Eq. **6**, it immediately follows that

[17] $$\begin{array}{c} Q_{d}^{\left(\alpha ,\beta \right)}\left(x\right)∼Cx^{-d-\alpha -\beta -1}\;,\;x\to \infty \end{array}$$

for some *C* ≠ 0, so $Q_{d}^{\left(\alpha ,\beta \right)}\left(x\right)=O\left(x^{-d-\alpha -\beta -1}\right)$ as x→∞. Now assume that *F* is given by Eq. **16** and satisfies $F\left(x\right)=O\left(x^{-d-\alpha -\beta -1}\right)$. Any polynomial can be written as a linear combination of Jacobi polynomials, thus

[18] $$\begin{array}{c} P\left(x\right)=\sum_{j=0}^{d}c_{j}P_{j}^{\left(\alpha ,\beta \right)}\left(x\right) \end{array}$$

for some $c_{j}\in R$. Consider

$$\begin{array}{c} G\left(x\right):=F\left(x\right)-2{\left(-1\right)}^{\alpha }\sum_{j=0}^{d}c_{j}Q_{j}^{\left(\alpha ,\beta \right)}\left(x\right)\;, \end{array}$$

where *c*_*j*_ are as in Eq. **18**. Then, on the one hand, Eqs. **15**, **16**, and **18** imply that G(x) is of the form ${\left(x-1\right)}^{-\alpha }{\left(x+1\right)}^{-\beta }\tilde{R}\left(x\right)$ for some polynomial $\tilde{R}$, and on the other hand, the assumption that $F\left(x\right)=O\left(x^{-d-\alpha -\beta -1}\right)$ and Eq. **17** imply that $G\left(x\right)=O\left(x^{-\alpha -\beta -1}\right)$. This can only happen if $\tilde{R}$ vanishes, and hence *G* = 0. Since $Q_{j}^{\left(\alpha ,\beta \right)}\left(x\right)∼C_{j}x^{-j-\alpha -\beta -1}$ for some $C_{j}\neq 0$ and $F\left(x\right)=O\left(x^{-d-\alpha -\beta -1}\right)$, we also see that $c_{j}=0$ for j=0,⋯,d−1, and thus, *F* is a multiple of $Q_{d}^{\left(\alpha ,\beta \right)}$, as claimed.

In view of the above characterization, the polynomial *R* from Eq. **15** can be computed from the following identity for formal Laurent series in *X*

$$\begin{array}{c} \begin{array}{cc} & \frac{{\left(1-X^{-1}\right)}^{\alpha }{\left(1+X^{-1}\right)}^{\beta }}{2}P_{d}^{\left(\alpha ,\beta \right)}\left(X^{-1}\right)\log(\frac{1+X}{1-X})\\ & \;=R\left(X^{-1}\right)+O\left(X\right)\;. \end{array} \end{array}$$

We note that each Jacobi function can be represented in terms of the hypergeometric function _2_*F*_1_. More precisely, from ref. 13, equation 4.61.5 and the symmetry

$$\begin{array}{c} Q_{d}^{\left(\alpha ,\beta \right)}\left(x\right)={\left(-1\right)}^{\alpha +\beta +d+1}Q_{d}^{\left(\beta ,\alpha \right)}\left(-x\right), \end{array}$$

we obtain

[19] $$\begin{array}{c} \begin{array}{cc} Q_{d}^{\left(\alpha ,\beta \right)}\left(x\right) & =\frac{2^{d+\alpha +\beta }\left(d+\alpha \right)!\left(d+\beta \right)!}{\left(2d+\alpha +\beta +1\right)!{\left(x-1\right)}^{\alpha }{\left(x+1\right)}^{d+\beta +1}}\\ & \;\times_{2}F_{1}(d+\beta +1,d+1;2d+\alpha +\beta +2;\frac{2}{1+x})\;, \end{array} \end{array}$$

where in order to incorporate the extension of $Q_{d}^{\left(\alpha ,\beta \right)}\left(x\right)$ to x∈(−1,1), we set for *t* > 1

[20] F21(a,b; c; t):=12(2F1(a,b; c; t+i0)+2F1(a,b; c; t−i0)).

We will use this definition of $Q_{d}^{\left(\alpha ,\beta \right)}\left(x\right)$ in terms of hypergeometric functions to compute Mellin transforms of Whittaker functions in the following section. Thus, we additionally need some results on the Mellin transform of the hypergeometric function _2_*F*_1_. Specifically, by (26, p. 314, equation 2.21.1.2),

[21] $$\begin{array}{c} \begin{array}{cc} & \int_{0}^{\infty }{\frac{\Gamma (a)\Gamma (b)\Gamma (c-a)\Gamma (c-b)}{\Gamma (c)}}_{2}F_{1}\left(a,b;c;1-x\right)x^{s-1}dx\\ & \;=\Gamma (s)\Gamma (s-(a+b-c))\Gamma (a-s)\Gamma (b-s)\;, \end{array} \end{array}$$

from which one also gets

[22] $$\begin{array}{c} \begin{array}{cc} & \int_{0}^{\infty }{\frac{\Gamma (a)\Gamma (b)\Gamma (c-a)\Gamma (c-b)}{\Gamma (c)}}_{2}F_{1}\left(a,b;\;c;\;1+x\right)x^{s-1}dx\\ & \;=\;\cos(\pi s)\Gamma (s)\Gamma (s-(a+b-c))\Gamma (a-s)\Gamma (b-s)\;, \end{array} \end{array}$$

where we define F21(a,b; c; t) for *t* > 1 as in Eq. **20**.

## 3. Proof of the Main Result

Let $W_{\kappa ,\mu }$ denote Whittaker’s *W*-functions as in ref. 27, Eq. 13.14.1 or (28, p. 386). It will be convenient to extend $W_{\kappa ,\mu }$ to a function defined on R∖{0} as follows

[23] $$\begin{array}{c} W_{\kappa ,\mu }\left(y\right):=\frac{\Gamma (1/2\;+\;\mu \;-\;\text{sgn}(y)\kappa )}{\Gamma (1/2\;+\;\mu -\kappa )}W_{\;\text{sgn}(y)\kappa ,\mu }\left(|y|\right)\;,\;y\neq 0\;. \end{array}$$

Next, we will need two results about $W_{\kappa ,\mu }$.

Lemma 1. *For* κ,μ≥0 *and* Re(s)>μ−1/2 *we have*

[24] $$\begin{array}{c} \int_{0}^{\infty }W_{\kappa ,\mu }\left(t\right)e^{-t/2}t^{s-1}dt=\frac{\Gamma (s-\mu +1/2)\Gamma (s+\mu +1/2)}{\Gamma (s-\kappa +1)}\; \end{array}$$

*and for* κ≥Re(s)>μ−1/2 *we have*

[25] $$\begin{array}{c} \begin{array}{cc} & \int_{0}^{\infty }W_{\kappa ,\mu }\left(-t\right)e^{t/2}t^{s-1}dt\\ & \;=\frac{\cos(\pi (\kappa -\mu ))}{\pi }\Gamma \left(s-\mu +1/2\right)\Gamma \left(s+\mu +1/2\right)\Gamma \left(\kappa -s\right)\;. \end{array} \end{array}$$

***Proof***: The first equation is a special case of (28, section 6.9 and Eq. **8**). The second equation is (28, section 6.9 and Eq. **7**) together with the functional equation for the gamma function. [Note that there is a typo in ref. 28, section 6.9 and Eq. **7**, compare with (27, Eq. 13.23.5).]

Lemma 2. *Let* $k_{1},k_{2},m_{1},m_{2}\in Z_{\geq 0}$ *satisfy* $m_{i}\leq k_{i}$ *and* $m_{1}+m_{2}<k_{1}+k_{2}$, *and set*

$$\ell :=k_{1}+k_{2}-m_{1}-m_{2}-1\;\;\text{and}\;q:=k_{1}+k_{2}+m_{1}+m_{2}-1.$$

*Then, for any* a,b∈R *such that* a+b=1 *and* ab≠0, *one has*

$$\begin{array}{c} \begin{array}{cc} \int_{0}^{\infty } & W_{k_{1},m_{1}}\left(ay\right)W_{k_{2},m_{2}}\left(by\right)e^{-y/2}y^{k_{1}+k_{2}-2}dy\\ & =\frac{2{\left(-1\right)}^{k_{1}-m_{1}}q!\ell !}{\pi }{\left|a\right|}^{m_{1}+1/2}{\left|b\right|}^{m_{2}+1/2}Q_{\ell }^{\left(2m_{1},2m_{2}\right)}\left(b-a\right)\;. \end{array} \end{array}$$

***Proof***: We first consider the case a∈(0,1) and define $I_{1}\left(x\right)$ for *x* > 0 by

$$\begin{array}{c} \begin{array}{c} x^{-m_{1}-1/2}\int_{0}^{\infty }W_{k_{1},m_{1}}\left(xy\right)W_{k_{2},m_{2}}\left(y\right)e^{-(1+x)y/2}y^{k_{1}+k_{2}-2}dy\;. \end{array} \end{array}$$

To prove the lemma in this case, it suffices to show that

[26] $$\begin{array}{c} I_{1}\left(x\right)=2{\left(-1\right)}^{k_{1}-m_{1}}\ell !q!\frac{Q_{\ell }^{\left(2m_{1},2m_{2}\right)}\left(\frac{1-x}{1+x}\right)}{\pi {\left(1+x\right)}^{q+1}}\;. \end{array}$$

Using [**24**] and (28, section 6.1 and Eq. **13**) we obtain that for 2m1<Re(s)<k1+k2+m1−m2, the Mellin transform of $I_{1}\left(x\right)$ is equal to

$$\begin{array}{cc} M(I_{1},s) & =\frac{\Gamma \left(s-2m_{1}\right)\Gamma \left(s\right)\Gamma \left(\ell +2m_{1}-s+1\right)\Gamma \left(q-s+1\right)}{\Gamma \left(s-m_{1}-k_{1}+1/2\right)\Gamma \left(k_{1}+m_{1}-s+1/2\right)}\\ & =\frac{1}{\pi }\cos\left(\pi (s-k_{1}-m_{1})\right)\Gamma \left(s-2m_{1}\right)\Gamma \left(s\right)\times \\ & \;\times \Gamma \left(\ell +2m_{1}-s+1\right)\Gamma \left(q-s+1\right). \end{array}$$

With the help of Eq. **19**, we write

[27] C1·Qℓ(2m1,2m2)(1−x1+x)(x+1)q+1=C1·Γq−2m2+1Γq−2m1+12Γ(2k1+2k2)×F21q−2m2+1,q+1;2k1+2k2;1+x.

By Eq. **22**, the Mellin transform of Eq. **27** is equal to

$$\begin{array}{cc} & C_{1}\cdot \frac{\cos\left(\pi s\right)\Gamma \left(s\right)\Gamma \left(s-2m_{1}\right)}{2\Gamma \left(\ell +1\right)\Gamma \left(q+1\right)}\\ & \;\times \Gamma \left(-s+q-2m_{2}+1\right)\Gamma \left(-s+q+1\right). \end{array}$$

In order for the expression above to coincide with $M(I_{1},s)$, we must require that

$$\begin{array}{c} C_{1}=\frac{2\Gamma \;\left(\ell +1\right)\Gamma \left(q+1\right)\;\cos\;\left(\pi \left(-k_{1}-m_{1}+s\right)\right)}{\pi \;\cos\;(\pi s)}. \end{array}$$

Thus, we obtain for $k_{1}+m_{1}\in Z$ that $I_{1}\left(x\right)$ is equal to

$$\begin{array}{c} \frac{2}{\pi }\Gamma \left(\ell +1\right)\Gamma \left(q+1\right)\cos\left(\pi \left(k_{1}+m_{1}\right)\right)\frac{Q_{\ell }^{\left(2m_{1},2m_{2}\right)}\left(\frac{1-x}{1+x}\right)}{{\left(x+1\right)}^{q+1}}. \end{array}$$

If $\ell ,q\in N_{0}$ and $k_{1}+m_{1}\equiv k_{1}-m_{1}\;mod\;2$, we get exactly [**26**].

It remains to prove the result for *a* < 0 (the case *b* < 0 follows by symmetry). For *x* > 0, we define $I_{2}\left(x\right)$ as

$$\begin{array}{c} \begin{array}{c} \int_{0}^{\infty }x^{-m_{1}-1/2}W_{k_{1},m_{1}}\left(-xy\right)W_{k_{2},m_{2}}\left(y\right)e^{-(1-x)y/2}y^{k_{1}+k_{2}-2}dy, \end{array} \end{array}$$

and again using *Lemma* 1 and (28, section 6.1 and Eq. **13**), we calculate $M(I_{2},s)$ is equal to

$$\begin{array}{cc} & \frac{\cos(\pi \left(k_{1}-m_{1}\right))}{\pi }\Gamma \left(s-2m_{1}\right)\Gamma \left(s\right)\Gamma \left(k_{1}+m_{1}-s+1/2\right)\\ & \;\times \frac{\Gamma \left(\ell +2m_{1}-s+1\right)\Gamma \left(q-s+1\right)}{\Gamma (k_{1}+m_{1}-s+1/2)} \end{array}$$

with the same fundamental strip as before. With the help of Eq. **19**, we write

[28] C2·Qℓ(2m1,2m2)(1+x1−x)(1−x)q+1=C2·Γq−2m2+1Γq−2m1+12Γ(2k1+2k2)×F21q−2m2+1,q+1;2k1+k2;1−x.

By Eq. **21**, the Mellin transform of Eq. **28** is equal to

$$\begin{array}{c} C_{2}\cdot \frac{\Gamma \left(s\right)\;\Gamma \left(s-2m_{1}\right)\;\Gamma \left(-s+q-2m_{2}+1\right)\;\Gamma \left(-s+q+1\right)}{2\;\Gamma \left(\ell +1\right)\;\Gamma \left(q+1\right)}. \end{array}$$

In order for the expression above to coincide with $M(I_{2},s)$, we must require that

$$\begin{array}{c} C_{2}=\frac{2}{\pi }\cos\left(\pi \left(k_{1}-m_{1}\right)\right)\;\Gamma \left(\ell +1\right)\;\Gamma \left(q+1\right). \end{array}$$

Thus, $I_{2}\left(x\right)$ is equal to

$$\begin{array}{c} \frac{2}{\pi }\cos\left(\pi \left(k_{1}-m_{1}\right)\right)\;\Gamma \left(\ell +1\right)\;\Gamma \left(q+1\right)\frac{Q_{\ell }^{\left(2m_{1},2m_{2}\right)}\left(\frac{1+x}{1-x}\right)}{{\left(1-x\right)}^{q+1}}. \end{array}$$

If we assume $k_{1}-m_{1}\in Z$ and $\ell ,q\in N_{0}$, then we obtain

[29] $$\begin{array}{c} I_{2}\left(x\right)=2{\left(-1\right)}^{k_{1}-m_{1}}\ell !q!\frac{Q_{\ell }^{\left(2m_{1},2m_{2}\right)}\left(\frac{1+x}{1-x}\right)}{\pi {\left(1-x\right)}^{q+1}}\;, \end{array}$$

which is easily seen to imply the claim of the lemma for *a* < 0.

Finally, we recall the holomorphic projection lemma from ref. 25 (we restrict to the case of ${\;\mathrm{SL}\;}_{2}\left(Z\right)$).

Lemma 3 [Holomorphic Projection Lemma]. *Let* $\tilde{\Phi }$ *be a nonholomorphic modular form of weight *k* > 2 for* ${\;\mathrm{SL}\;}_{2}\left(Z\right)$ *with a Fourier expansion* $\tilde{\Phi }\left(z\right)=\sum_{m\in Z}a_{m}\left(y\right)e^{2\pi imx}$, *and suppose that for some *ε* > 0 we have* $\tilde{\Phi }\left(z\right)=O\left(y^{-\varepsilon }\right)$ *as* z→i∞. *Define*

$$\begin{array}{c} a_{m}=\frac{{\left(4\pi m\right)}^{k-1}}{(k-2)!}\int_{0}^{\infty }a_{m}\left(y\right)e^{-2\pi my}y^{k-2}dy\;,\;m>0\;. \end{array}$$

*Then, the function* $\Phi \left(z\right)=\sum_{m>0}a_{m}e^{2\pi imz}$ *is a holomorphic cusp form of weight *k* for* ${\;\mathrm{SL}\;}_{2}\left(Z\right)$ *and moreover* $\left\langle f,\Phi \right\rangle =\left\langle f,\tilde{\Phi }\right\rangle$ *for all* $f\in S_{k}\left({\;\mathrm{SL}\;}_{2}\left(Z\right)\right)$.

***Proof of Theorem 1***: With our notation for $W_{\kappa ,\mu }$ the completed nonholomorphic Eisenstein series $E_{2k}^{*}\left(z,s\right)$, $k\in Z_{\geq 0}$, has the following Fourier expansion [cf. (29, p. 210), (30, section 3)]

$$\begin{array}{c} E_{2k}^{*}\left(z,s\right)=c_{k,s}\left(y\right)+{\left(-1\right)}^{k}\sum_{n\neq 0}\frac{\sigma_{2s-1}\left(n\right)}{{\left|n\right|}^{s}}W_{k,s-1/2}\left(4\pi ny\right)e^{2\pi \;inx}\;. \end{array}$$

Here,

$$\begin{array}{c} c_{k,s}\left(y\right)=\left\{\begin{array}{c} \theta_{k}\left(s\right)y^{s}+\theta_{k}\left(1-s\right)y^{1-s}\\ \;\;\;\;\;\;\;\;\;\;\;\;\;\;\;\;\;\;\;\;\;\;\;\;\;\;\;\;\;\;\;\;\;\;\text{for}\;s>1/2\;,\\ \frac{\Gamma \left(k+\frac{1}{2}\right)}{\sqrt{\pi }}\left(\psi \left(k+\frac{1}{2}\right)+2\gamma +\log\left(y/\pi \right)\right)\sqrt{y}\\ \;\;\;\;\;\;\;\;\;\;\;\;\;\;\;\;\;\;\;\;\;\;\;\;\;\;\;\;\;\;\;\;\;\;\text{for}\;s=1/2\;, \end{array}\right. \end{array}$$

where $\theta_{k}\left(s\right)=\pi^{-s}\Gamma \left(s+k\right)\zeta \left(2s\right)$, ψ(z) is the digamma function, and *γ* denotes the Euler–Mascheroni constant. Denote $m_{i}=r_{i}/2$ and let

$$\begin{array}{c} \tilde{\Phi }\left(z\right)=E_{2k_{1}}^{*}\left(z,m_{1}+1/2\right)E_{2k_{2}}^{*}\left(z,m_{2}+1/2\right)y^{-k_{1}-k_{2}}, \end{array}$$

where we choose the integers *k*_1_ and *k*_2_ to satisfy $2k_{1}+2k_{2}=k=r_{1}+r_{2}+2d+2$ and $k_{i}\geq m_{i}$. Expanding the product of the Fourier expansions of the two Eisenstein series we see that the coefficients of the holomorphic projection of $\tilde{\Phi }$ can be calculated as

$$\begin{array}{c} a_{n}=\sum_{n_{1}+n_{2}=n}a_{n_{1},n_{2}}\;, \end{array}$$

where for $n_{1}n_{2}\neq 0$ we have

$$\begin{array}{cc} a_{n_{1},n_{2}} & =\frac{{\left(4\pi n\right)}^{w-1}}{(w-2)!}\frac{{\left(-1\right)}^{k_{1}+k_{2}}\sigma_{r_{1}}\left(n_{1}\right)\sigma_{r_{2}}\left(n_{2}\right)}{|n_{1}{|}^{m_{1}+1/2}{\left|n_{2}\right|}^{m_{2}+1/2}}\\ & \;\times \int_{0}^{\infty }W_{k_{1},m_{1}}\left(4\pi n_{1}y\right)W_{k_{2},m_{2}}\left(4\pi n_{2}y\right)e^{-2\pi ny}y^{k_{1}+k_{2}-2}dy\\ & =2{\left(-1\right)}^{k_{2}+m_{1}}{\left(4\pi \right)}^{k_{1}+k_{2}}n^{d}\sigma_{r_{1}}\left(n_{1}\right)\sigma_{r_{2}}\left(n_{2}\right)\\ & \;\times \frac{d!(d+r_{1}+r_{2})!}{\pi (2d+r_{1}+r_{2})!}Q_{d}^{\left(r_{1},r_{2}\right)}(\frac{n_{2}-n_{1}}{n}) \end{array}$$

by *Lemma* 2. The boundary terms can be calculated from *Lemma* 1 as

[30] $$\begin{array}{c} \begin{array}{cc} a_{n,0} & ={\left(-1\right)}^{k_{2}}\frac{{\left(4\pi n\right)}^{k-1}}{(k-2)!}\frac{\sigma_{r_{1}}\left(n\right)}{{\left|n\right|}^{m_{1}+1/2}}\\ & \;\times \int_{0}^{\infty }W_{k_{1},m_{1}}\left(4\pi ny\right)c_{k_{2},m_{2}+1/2}\left(y\right)e^{-2\pi ny}y^{k_{1}+k_{2}-2}dy, \end{array} \end{array}$$

that is,

$$\begin{array}{c} \begin{array}{cc} a_{n,0} & ={\left(-1\right)}^{k_{2}}\frac{2^{k}\pi^{\frac{k}{2}-1}\cos\left(\pi m_{2}\right)}{\left(k-2\right)!}n^{d}\sigma_{r_{1}}\left(n\right)\\ & \;\times \;(n^{r_{2}}\zeta \left(r_{2}\right)\Gamma \left(r_{2}\right)\Gamma \left(d+1\right)\Gamma \left(\frac{k}{2}+m_{1}-m_{2}\right)\\ & \;+\;\zeta \left(-r_{2}\right)\Gamma \left(-r_{2}\right)\Gamma \left(\frac{k}{2}-m_{1}+m_{2}\right)\\ & \;\times \;\Gamma \left(r_{1}+r_{2}+d+1\right)), \end{array} \end{array}$$

where $r_{2}\neq 0$ and, similarly,

[31] $$\begin{array}{cc} a_{0,n} & ={\left(-1\right)}^{k_{1}}\frac{2^{k}\pi^{\frac{k}{2}-1}\cos\left(\pi m_{1}\right)}{\left(k-2\right)!}n^{d}\sigma_{r_{2}}\left(n\right)\\ & \;\times \;(n^{r_{1}}\zeta \left(r_{1}\right)\Gamma \left(r_{1}\right)\Gamma \left(d+1\right)\Gamma \left(\frac{k}{2}-m_{1}+m_{2}\right)\\ & \;+\;\zeta \left(-r_{1}\right)\Gamma \left(-r_{1}\right)\Gamma \left(\frac{k}{2}+m_{1}-m_{2}\right)\\ & \;\times \;\Gamma \left(r_{1}+r_{2}+d+1\right)) \end{array}$$

when $r_{1}\neq 0$. When $r_{2}=0$, $a_{n,0}$ can be obtained by taking a limit $r_{2}\to 0$ in Eq. **30** due to the absolute convergence of the respective integrals:

$$\begin{array}{cc} a_{n,0} & =\frac{{\left(-1\right)}^{k_{2}}2^{k-1}\pi^{\frac{k}{2}-1}n^{\frac{k}{2}-m_{1}-1}\Gamma \left(\frac{k}{2}-m_{1}\right)\Gamma \left(\frac{k}{2}+m_{1}\right)}{\left(k-2\right)!}\\ & \;\times \left(H_{k/2-m_{1}-1}+H_{k/2+m_{1}-1}-\log\left(4\pi^{2}n\right)\right)\sigma_{r_{1}}\left(n\right), \end{array}$$

where $H_{d}=\psi \left(d+1\right)+\gamma$ and

$$\begin{array}{cc} a_{0,n} & =\frac{4^{d+m_{1}+1}\pi^{d+m_{1}}\cos\left(\pi m_{1}\right)n^{d-m_{1}}\sigma_{0}\left(n\right)}{\left(2\left(d+m_{1}\right)\right)!}\\ & \;\times (\Gamma {\left(d+1\right)}^{2}n^{2m_{1}}\zeta \left(r_{1}\right)\Gamma \left(2m_{1}\right)\\ & \;+\zeta \left(-r_{1}\right)\Gamma \left(-r_{1}\right)\Gamma {\left(d+2m_{1}+1\right)}^{2}) \end{array}$$

Similarly, when $r_{1}=0$, $a_{0,n}$ can be obtained by taking a limit $r_{1}\to 0$ in Eq. **31**. When $r_{1}=r_{2}=0$, we obtain

$$\begin{array}{c} \begin{array}{cc} a_{0,n}=a_{n,0} & =\frac{2^{2d+1}{\left(-1\right)}^{d}\pi^{d}n^{d}\Gamma {\left(d+1\right)}^{2}}{(2d)!}\\ & \;\times \left(2H_{d}-\log\left(4\pi^{2}n\right)\right)\sigma_{0}\left(n\right). \end{array} \end{array}$$

By the result of Diamantis and O’Sullivan (31, Prop. 2.1), for any normalized Hecke eigenform $f\in S_{k}\left({\;\mathrm{SL}\;}_{2}\left(Z\right)\right)$ we have

$$\begin{array}{cc} \left\langle f,\tilde{\Phi }\right\rangle & =2{\left(-1\right)}^{k_{2}}\pi^{k}L^{⋆}\left(f,d+1\right)L^{⋆}\left(f,r_{2}+d+1\right)\\ & \;=2{\left(-1\right)}^{k_{2}+m_{2}-m_{1}-d-1}\pi^{k}L^{⋆}\left(f,d+1\right)L^{⋆}\left(f,r_{1}+d+1\right)\;, \end{array}$$

from which, in combination with the above formulas for $a_{n_{1},n_{2}}$, we recover [**7**].

***Proof of Corollary 1***: We recall *n* > 0. We use the substitution $n_{1}=\frac{n}{2}\left(1-x\right)$, $n_{2}=\frac{n}{2}\left(1+x\right)$ so that $\varphi (n_{1},n_{2})$ becomes a function of *n* and *x*, call it $\varphi_{n}\left(x\right)$. The summation in *φ*_*n*_ containing logarithms becomes

$$\begin{array}{c} \log|\frac{x+1}{x-1}|P_{n,2}\left(x\right)+\log\left|x-1\right|\left(P_{n,2}\left(x\right)+P_{n,3}\left(x\right)\right), \end{array}$$

where $P_{n,2}\left(x\right)$ and $P_{n,3}\left(x\right)$ are polynomials in *x* with the degree at most *d*. The growth condition $\varphi \left(n_{1},n-n_{1}\right)=O\left(n_{1}^{-d-r_{1}-r_{2}-1}\right)$ for $n_{1}\to \pm \infty$ and fixed *n* implies that $P_{n,2}\left(x\right)+P_{n,3}\left(x\right)$ vanishes. After substitution, we get

$$\varphi_{n}\left(x\right)=\frac{P_{n,1}\left(x\right)}{{\left(1-x\right)}^{r_{1}}{\left(1+x\right)}^{r_{2}}}+P_{n,2}\left(x\right)\log\left|\frac{x+1}{x-1}\right|,$$

where $P_{n,1}\left(x\right)$ is a polynomial in *x* of degree at most $r_{1}+r_{2}+d-1$. Thus, by *Proposition* 1, the function $\varphi_{n}\left(x\right)$ (considered as a function of *x* while keeping *n* fixed) is a constant multiple of $Q_{d}^{\left(r_{1},r_{2}\right)}\left(x\right)$. We recall that the coefficient of $Q_{d}^{\left(r_{1},r_{2}\right)}\left(\frac{n_{2}-n_{1}}{n_{1}+n_{2}}\right)$ in front of $\log|n_{1}|$ is equal to

$$\begin{array}{c} \begin{array}{cc} & \frac{{\left(-1\right)}^{r_{1}+1}}{2}P_{d}^{\left(r_{1},r_{2}\right)}\left(\frac{n_{2}-n_{1}}{n_{1}+n_{2}}\right)\\ & \;=\frac{{\left(-1\right)}^{r_{1}+1}}{2{\left(n_{1}+n_{2}\right)}^{d}}\sum_{s=0}^{d}\frac{{\left(-1\right)}^{s}\left(d+r_{1}\right)!\left(d+r_{2}\right)!n_{1}^{s}n_{2}^{d-s}}{s!\left(d-s\right)!\left(s+r_{1}\right)!\left(d-s+r_{2}\right)!}. \end{array} \end{array}$$

Changing the variables $n_{2}=n-n_{1}$, we obtain a polynomial in $\frac{n_{1}}{n}$ with leading coefficient

$$\begin{array}{cc} & \frac{{\left(-1\right)}^{r_{1}+1}}{2}\sum_{s=0}^{d}{\left(-1\right)}^{d}\frac{\left(d+r_{1}\right)!\left(d+r_{2}\right)!}{s!\left(d-s\right)!\left(s+r_{1}\right)!\left(d-s+r_{2}\right)!}\\ & \;=\frac{{\left(-1\right)}^{r_{1}+d+1}}{2}\frac{(r_{1}+\beta +2d)!}{d!(r_{1}+r_{2}+d)!}{\left(\frac{n_{1}}{n}\right)}^{d}. \end{array}$$

Comparing the leading coefficients of $\varphi_{n}\left(x\right)$ and $Q_{d}^{\left(r_{1},r_{2}\right)}\left(x\right)$ we see that

$$\varphi_{n}\left(x\right)={\left(-1\right)}^{r_{1}+d+1}C_{d}n^{d}\frac{2d!(r_{1}+r_{2}+d)!}{(r_{1}+r_{2}+2d)!}Q_{d}^{\left(r_{1},r_{2}\right)}\left(x\right).$$

The expression above also fixes *A*_*j*_, *B*_*j*_, *C*_*j*_, and *D*_*j*_.

## Data Availability

There are no data underlying this work.

## Footnotes

^*^We say that a Hecke eigenform is normalized if its first nonzero Fourier coefficient is equal to 1.

^†^We were unable to find a primary source for this result.

^‡^e.g., in the first case, $r_{1}=r_{2}=2,d=3$, and thus, $k=2d+r_{1}+r_{2}+2=12$.

## References

[1] S. Ramanujan, Collected Papers (Chelsea, 1962).

[2] S. Baluyot, C. L. Turnage-Butterbaugh, Twisted 2*k*th moments of primitive Dirichlet *L*-functions: Beyond the diagonal. arXiv [Preprint] (2022). https://arxiv.org/abs/2205.00641 (Accessed 5 October 2024).

[3] V. Blomer, Shifted convolution sums and subconvexity bounds for automorphic *L*-functions. Int. Math. Res. Not. **2004**, 3905–3926 (2004).

[4] J. B. Conrey, D. W. Farmer, J. P. Keating, M. O. Rubenstein, N. C. Snaith, Integral moments of *L*-functions. Proc. Lond. Math. Soc. **91**, 33–104 (2005).

[5] L. F. Alday, S. M. Chester, D. Dorigoni, M. B. Green, C. Wen, Relations between integrated correlators in N = 4 supersymmetric Yang-Mills Theory. J. High Energy Phys. 2024.5, 1–44 (2024).

[6] S. M. Chester, M. B. Green, S. S. Pufu, Y. Wang, C. Wen, New modular invariants in N = 4 Super-Yang-Mills theory. J. High Energy Phys. **2021**, 1–58 (2021).35342281

[7] D. Dorigoni, R. Treilis, Two string theory flavors of generalized Eisenstein series. J. High Energy Phys. 2023.11, 1–54 (2023).

[8] M. Green, S. D. Miller, J. G. Russo, P. Vanhove, Eisenstein series for higher-rank groups and string theory amplitudes. Commun. Number Theory Phys. **4**, 551–596 (2010).

[9] M. Green, S. D. Miller, P. Vanhove, SL(2,Z)-invariance and D-instanton contributions to the $D^{6}R^{4}$ interaction Commun. Number Theory Phys. **9**, 307–344 (2015).

[10] M. Green, J. G. Russo, P. Vanhove, Automorphic properties of low energy string amplitudes in various dimensions. Phys. Rev. D **81**, 086008 (2010).

[11] K. Klinger-Logan, S. D. Miller, D. Radchenko, The $D^{6}R^{4}$ interaction as a Poincaré series, and a related shifted convolution sum. arXiv [Preprint] (2022). https://arxiv.org/abs/2210.00047 (Accessed 5 October 2024).

[12] A. Erdélyi , Higher Transcendental Functions (McGraw-Hill, 1953), vol. II.

[13] G. Szegö, *Orthogonal Polynomials* (American Mathematical Society, New York, 1959).

[14] K. Fedosova, K. Klinger-Logan, Whittaker Fourier type solutions to differential equations arising from string theory. Commun. Number Theory Phys. **17**, 583–641 (2023).

[15] J. W. L. Glaisher, “Expressions for the first five powers of series in which the coefficients are the sums of the divisors of the exponents” in *Mathematical Papers, Chiefly Connected with the Q-Series in Elliptic Functions*, J. W. L. Glaisher, Ed. (W. Metcalfe and Son, Cambridge, 1885).

[16] Y. Motohashi, The binary additive divisor problem. Ann. Sci. Éc. **27**, 529–572 (1994).

[17] D. Lahiri, Identities connecting elementary divisor functions of different degrees, and allied congruences. Math. Scand. **24**, 102–110 (1969).

[18] J. H. Brinier, G. van der Geer, G. Harder, D. Zagier, 1-2-3 Modular Forms: Lectures at a Summer School in Nordfjordeid, Norway (Springer, 2008).

[19] C. O’Sullivan, “Identities from the holomorphic projection of modular forms” in *Number Theory for the Millennium*, B. Berndt et al., Eds. (Routledge, 2002), vol. 3, pp. 87–106.

[20] N. Diamantis, Kernels of *L*-functions and shifted convolutions. Proc. Am. Math. Soc. **148**, 5059–5070 (2020).

[21] K. Fedosova, K. Klinger-Logan, Shifted convolution sums motivated by string theory. J. Number Theory **260**, 151–172 (2024).

[22] D. J. Binder, S. M. Chester, S. S. Pufu, Y. Wang, N = 4 Super-Yang-Mills correlators at string coupling from string theory and localization. JHEP **12**, 119 (2019).

[23] S. M. Chester, S. S. Pufu, Far beyond the planar limit in strongly coupled N = 4 sym. JHEP **01**, 103 (2021).

[24] Y. I. Manin, Periods of parabolic forms and p-adic Hecke series. Mat. Sb. **134**, 378–401 (1973).

[25] B. Gross, D. Zagier, Heegner points and derivatives of *L*-series. Invent. Math. **84**, 225–320 (1986).

[26] A. P. Prudnikov , Integrals and Series (Gordon & Breach Sci. Publ., 1986), vol. III.

[27] NIST Digital Library of Mathematical Functions (2023). https://dlmf.nist.gov/ Release 1.2.2 of 2024-09-15. Accessed 15 September 2023.

[28] A. Erdélyi , Tables of Integral Transforms (McGraw-Hill, 1954), vol. I.

[29] H. Maass, *Lectures on Modular Functions of One Complex Variable* (Tata Institute of Fundamental Research, Bombay, ed. 2, 1983). vol. 29.

[30] C. O’Sullivan, Formulas for non-holomorphic Eisenstein series and for the Riemann zeta function at odd integers. Res. Number Theory **4**, 36 (2018).

[31] N. Diamantis, C. O’Sullivan, Kernels of *L*-functions of cusp forms. Math. Ann. **346**, 897–929 (2010).

